# Logistic regression model for the prediction of asthenia development in schizophrenia based on inflammatory blood markers

**DOI:** 10.1192/j.eurpsy.2024.586

**Published:** 2024-08-27

**Authors:** S. A. Zozulya, A. N. Simonov, T. P. Klyushnik

**Affiliations:** Mental Health Research Centre, Moscow, Russian Federation

## Abstract

**Introduction:**

According to a number of authors, inflammation is involved in the development of asthenic syndrome in different diseases. The results of our own studies indicate that the main feature of the spectrum of inflammatory markers in patients with asthenic syndrome in schizophrenia is low enzymatic activity of leukocyte elastase against the background of high levels of other inflammatory markers. Presumably, the decrease in LE activity may be associated with functional exhaustion of neutrophils and/or their transmigration to the brain through the disrupted blood-brain barrier due to a long-term chronic inflammatory process.

**Objectives:**

To create a logistic regression model for predicting the development of asthenia in schizophrenia based on the analysis of the association between leukocyte elastase (LE) and α1-proteinase inhibitor (α1-PI) activity in blood plasma.

**Methods:**

A database including clinical and demographic parameters (ICD-10 diagnosis, duration of the disease, psychometric evaluation according to the PANSS and MFI-20 scales, sex and age) and immunological parameters (enzymatic activity of LE and functional activity of α1-PI in blood plasma) of 95 patients from 22 to 55 years old with paroxysmal-progressive (F20.x1) and paranoid (F20.00) schizophrenia was used to construct the model. An asthenic symptom complex was diagnosed in 61 patients.

**Results:**

A binary logistic regression model linking the probability of developing asthenia to LE and α1-PI activity was constructed by analyzing a database of patients with schizophrenia.

P=1/(1+exp [-(11.71-0.057-LE+0.027-α1-PI)] (2), where

P is the probability of asthenia development; exp is the base of the natural logarithm; 11.71 is the regression constant; 0.057 is the coefficient for LE; 0.027 is the coefficient for α1-PI.

This model adequately describes the clinical data and has good predictive ability (sensitivity - 93.44%, specificity - 76.47%, AUC - 0.89).

**Conclusions:**

A binary logistic regression model was created to predict the development of asthenia in schizophrenia using immunological parameters LE and α1-PI. The model is highly effective and can complement clinical examination of patients with schizophrenia, contributing to the objective diagnosis of asthenic syndrome and, consequently, timely therapeutic correction.

**Disclosure of Interest:**

None Declared

